# Editorial Notes

**Published:** 1894-07

**Authors:** 


### Editorial Notes.

---

Dr. J. B. S. Holmes has been elected Assistant Professor of Obstetrics and Gynecology in Southern Medical College.

---

The first issue of *The Southern Medical Review*, edited and published by Dr. N. J. Phenix, of Houston, Texas, is to hand. It is a handsome and well edited issue, and we extend to it a hearty welcome.

---

Mr. R. E. Queen, of San Francisco, tendered a banquet to the visiting members of the American Medical Editors' Association on June 4th. An elegant menu was served, and was, of course, highly appreciated and enjoyed by the visitors.

---

In the July issue of the *Journal of Nervous and Mental Diseases*, of New York, will appear the address of Dr. S. Weir Mitchell, to the American Medico-Psychological Association. No physician can afford to miss this valuable address.

---

Dr. W. E. Campbell, late of New York, has entered into a partnership with Dr. George Brown, the Eye, Ear, Nose and Throat Specialist of this city. Dr. Campbell is a polished, refined gentleman, and we wish the new firm abundant success.

---

In our last number we stated that Dr. R. O. Cotter, of Macon, would remain abroad two years. This is a mistake, as Dr. Cotter will be home by October next. A year and a half ago Hon. R. J. Powell, of Barnesville, President of the Barnesville Savings Bank, died. As Dr. Cotter and his family were both largely interested in this bank, upon Captain Powell's death Dr. Cotter, being Vice-President of the bank, was elected President, and has had the management of the bank upon his hands ever since, so that he has had for a time practically to abandon his profession. He will now turn over the management of the business to other hands, and after spending four months brushing up in the eye, ear and throat hospitals of New York and Europe, he will resume his practice in the fall. The doctor will write us a letter every month while away.

